# Targeting Notch and EGFR signaling in human mucoepidermoid carcinoma

**DOI:** 10.1038/s41392-020-00388-0

**Published:** 2021-01-21

**Authors:** Wei Ni, Zirong Chen, Xin Zhou, Rongqiang Yang, Mu Yu, Jianrong Lu, Frederic J. Kaye, Lizi Wu

**Affiliations:** 1 Department of Molecular Genetics and Microbiology, University of Florida, Gainesville, FL 32610 USA; 2 UF Health Cancer Center, University of Florida, Gainesville, FL 32610 USA; 3 UF Genetics Institute, University of Florida, Gainesville, FL 32610 USA; 4 Department of Biochemistry and Molecular Biology, University of Florida, Gainesville, FL 32610 USA; 5 Department of Medicine, University of Florida, Gainesville, FL 32610 USA

**Keywords:** Cancer stem cells, Cancer therapy, Head and neck cancer

## Abstract

Mucoepidermoid carcinoma (MEC) is the most common type of salivary gland cancers and patients with advanced, metastatic, and recurrent MECs have limited therapeutic options and poor treatment outcomes. MEC is commonly associated with a chromosomal translocation t(11;19) (q14-21;p12-13) that encodes the CRTC1-MAML2 oncogenic fusion. The CRTC1-MAML2 fusion is required for MEC growth in part through inducing autocrine AREG-EGFR signaling. Growing evidence suggests that MEC malignancy is maintained by cancer stem-like cells. In this study, we aimed to determine critical signaling for maintaining MEC stem-like cells and the effect of combined targeting of stem cell signaling and CRTC1-MAML2-induced EGFR signaling on blocking MEC growth. First, we evaluated the significance of Notch signaling in regulating MEC stem-like cells. Aberrantly activated Notch signaling was detected in human fusion-positive MEC cells. The inhibition of Notch signaling with genetic or pharmacological inhibitors reduced oncosphere formation and ALDH-bright population in vitro and blocked the growth of MEC xenografts in vivo. Next, we investigated the effect of co-targeting Notch signaling and EGFR signaling, and observed enhanced inhibition on MEC growth in vivo. Collectively, this study identified a critical role of Notch signaling in maintaining MEC stem-like cells and tumor growth, and revealed a novel approach of co-targeting Notch and EGFR signaling as a potential effective anti-MEC treatment.

## Introduction

Mucoepidermoid carcinoma (MEC) is the most common salivary gland malignancy and can also develop in multiple other sites.^1,2^ MEC is histologically characterized by the presence of three different cell types including epidermoid cells, mucin-secreting cells, and intermediate cells. While low-grade MECs are usually cured by surgical resection, recurrent or advanced MECs are associated with unfavorable outcomes. Currently, there is a lack of effective systemic treatment options for patients with MEC.

MEC is frequently associated with a unique chromosomal translocation t(11;19) (q14-21;p12-13) that creates the CRTC1-MAML2 fusion. The *CRTC1-MAML2* fusion transcripts have been detected in up to 80% of human MEC tumors in several MEC cohorts.^3–6^ The *CRTC1-MAML2* fusion protein consists of the 42-aa CREB binding domain (CBD) of the CREB transcriptional co-activator CRTC1 at its N terminus and the 981-aa transcriptional activation domain (TAD) of the Notch transcriptional co-activator MAML2 at its C terminus.^7^ The CRTC1-MAML2 fusion was capable of transforming epithelial cells and its knockdown reduced the growth and survival of human MEC cells,^7–11^ supporting its role as an oncogenic driver in MEC development and maintenance. Mechanistically, a major action of the CRTC1-MAML2 fusion is to interact with CREB and aberrantly activate a CREB-mediated transcriptional program that promotes its oncogenic activity.^9,10,12^ In addition, this fusion interacted and activated MYC and AP-1.^13,14^

The CRTC1-MAML2 fusion is a potential therapeutic target as MEC cells depend on its expression for growth and survival.^11^ This fusion protein is localized in the nucleus and has no known enzymatic activity;^9^ so it is traditionally difficult to target. Significant efforts have been directed into identifying critical signaling pathways downstream of the CRTC1-MAML2 fusion in order to uncover therapeutic approaches.^9–12,15^ For instance, we have shown that the CRTC1-MAML2 fusion upregulates the expression of amphiregulin (AREG), an EGFR ligand via co-activating the transcription factor CREB and consequently inducing EGFR signaling in an autocrine manner.^11^ As a result, human fusion-positive MEC cells were highly sensitive to EGFR signaling inhibition, demonstrated by the observation that the EGFR monoclonal antibody Cetuximab significantly inhibited MEC cell growth in vitro and in vivo.^11^ However, EGFR inhibition was unable to eradicate all the MEC cells and a small population of surviving cells persisted. Moreover, resistance is commonly associated with the use of EGFR inhibitors in cancer patients in clinic.^16^ Therefore, strategies for blocking additional signaling critical for tumor growth likely lead to enhanced anti-tumor responses and reduced tumor resistance.

MEC displays striking cellular heterogeneity. MEC shares similar cytokeratin expression profiles with normal salivary gland stem cells and contains a small population of cells expressing specific stem cell markers and exhibiting highly tumorigenic ability.^17–22^ Moreover, MEC is resistant to chemoradiotherapy.^23,24^ These lines of evidence strongly suggest that MEC arises from the transformation of salivary gland stem/progenitor cells and is maintained by MEC stem-like or tumor-initiating cells. However, the molecular regulation of MEC stem-like cells remained poorly characterized.

The Notch signaling pathway is evolutionarily conserved and important in multiple developmental processes and diseases.^25,26^ In mammalian cells, Notch cell-surface receptors (Notch 1, 2, 3, 4) transduce intercellular communications by interacting with the transmembrane ligands (Delta-like 1, 3, 4 and Jagged 1, 2) on neighboring cells. Ligand binding triggers proteolytic cleavages of Notch receptors, including ADAM-mediated S2 cleavage and the subsequent γ-secretase-mediated S3 cleavage, leading to the release of the intracellular domain of Notch receptors (ICN) from the cell membrane. ICN then travels to the nucleus and forms the Notch transcriptional core complex with the transcription factor CSL and the family of three transcriptional MAML coactivators, thereby activating the transcription of Notch target genes.^27,28^ Notch signaling has been shown to critically regulate multiple normal and cancerous stem cells.^29–36^ However, whether Notch signaling is important in regulating MEC stem-like cells has not yet been investigated.

In this study, we evaluated the functional role of Notch signaling in human MEC. We subsequently assessed the anti-tumor responses of co-targeting Notch signaling that maintains MEC stem-like cells and EGFR signaling downstream of the CRTC1-MAML2 oncogenic fusion in MEC. Our data demonstrated that Notch signaling plays a critical role in maintaining MEC stem-like cells and MEC tumor growth and revealed that a novel approach of targeting Notch and EGFR signaling serves as a potential effective anti-MEC treatment.

## Results

### NOTCH1 signaling was activated in human CRTC1-MAML2 fusion-positive MEC cells

**Fig. 1:**
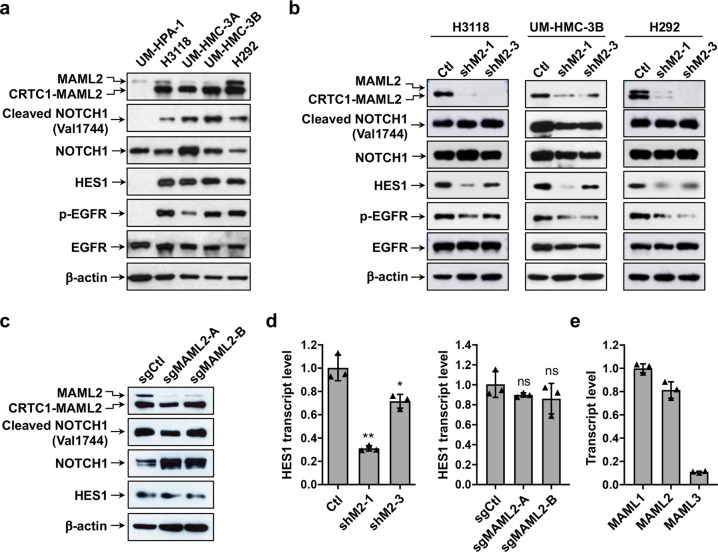
NOTCH1 and EGFR signaling pathways were activated in human CRTC1-MAML2 fusion-positive MEC cells. **a** Western blot analysis was carried out on human CRTC1-MAML2-positive MEC cell lines (H3118, UM-HMC-3A, UM-HMC-3B, and H292) and a fusion-negative cells line (UM-HPA-1) using various antibodies as indicated. **b** Human MEC cells (H3118, UM-HMC-3B, and H292) were transduced with lentiviruses expressing shRNAs (shM2-1, shM2-3) that target the respective 3′ UTR and TAD domain of the CRTC1-MAML2 fusion and MAML2, or control shRNA (Ctl). Cells were collected at 96 h after viral infection and western blotting was performed. **c** Stable Cas9-expressing H3118 cells were transduced with lentiviruses expressing sgRNAs that target the exon 1 of MAML2 (sgMAML2-A, sgMAML2-B) or control (sgCtl). Cells were harvested at 96 h after viral infection and western blotting was performed. **d** Quantitative RT-PCR analysis of HES1 transcript levels in transduced MEC H3118 cells as described in (**b**) and (**c**) (*n* = 3; ns, *p* > 0.05; *p < 0.05; **p < 0.01). **e** Quantitative RT-PCR analysis of the transcript levels of three MAML co-activator family gene members, *MAML1*, *MAML2*, and *MAML3* in human MEC H3118 cells

To investigate whether Notch signaling plays a role in regulating MEC, we first determined the Notch signaling status in four human CRTC1-MAML2 fusion-positive MEC cell lines, including H292 (metastatic pulmonary MEC), H3118 (metastatic parotid MEC), UM-HMC-3A and UM-HMC-3B (from a local recurrence and metastatic lymph node from the same patient with MEC, respectively), and a fusion-negative cell line UM-HPA-1 (benign pleomorphic adenocarcinoma). We detected the presence of the cleaved form of NOTCH1 (Val1744), indicative of activated NOTCH1 receptor, and expression of *HES1*, a Notch signaling-induced downstream target in all four human CRTC1-MAML2 fusion-positive MEC cell lines but not in fusion-negative cells (Fig. 1a).

We next determined whether Notch signaling activation is caused by the CRTC1-MAML2 fusion. Since we failed to obtain fusion-specific shRNAs, we compared the effect of the depletion of both the CRTC1-MAML2 fusion and MAML2 with two independent lentivirus-mediated shRNAs (shM2-1 and shM2-3 that target the respective 3′ UTR and TAD domain sequences of the CRTC1-MAML2 fusion and MAML2) (Fig. 1b) and that of Crispr/cas9-mediated knockout of MAML2 using two single-guide RNAs (sgMAML2-A and sgMAML2-B that target the exon 1 of MAML2) in order to identify fusion-specific activity in MEC (Fig. 1c). MEC cells (H3118, UM-HMC-3B, and H292) transduced with these two shRNAs (shM2-1 and shM2-3) had reduced EGFR signaling, as shown by the decreased phosphorylated EGFR (p-EGFR) level (Fig. 1b), which is consistent with the previous finding that the CRTC1-MAML2 fusion induces EGFR signaling.^11^ We observed little or no change in the cleaved NOTCH1 level (Fig. 1b), but decreased HES1 expression at the transcript and protein levels in MEC cells depleted of the fusion/MAML2 as compared with cells expressing scrambled shRNA control (Ctl) (Fig. 1b, d). MAML2 knockout (sgMAML2-A and sgMAML2-B) did not reduce cleaved NOTCH1 level (Fig. 1c) and HES1 expression at the protein and transcript levels in comparison with the control (sgCtl) (Fig. 1c, d). The CRTC1-MAML2 regulation of HES1 expression is consistent with the previous studies showing that the Notch target *HES1* gene promoter can also be activated by the CRTC1-MAML2 fusion through a CRE site in its promoter.^7,37^ Therefore, the depletion of the CRTC1-MAML2 fusion did not impact the Notch receptor activation, but reduced HES1 expression.

Furthermore, we observed all three MAML co-activator members are all expressed in human MEC cells at various levels (Fig. 1e), which suggests their potential functional redundancy in activating Notch-mediated transcription and likely explains why the knockout of the MAML2 member alone had no effect on the HES1 expression. Collectively, our data showed that Notch receptor signaling is activated in human MEC cells, which is independent of the CRTC1-MAML2 fusion expression. Moreover, expression of the Notch target gene *HES1* is also regulated by the CRTC1-MAML2 fusion in MEC cells.

### Expression of a pan-Notch inhibitor, a dominant-negative MAML1 mutant, reduced MEC oncosphere-forming and ALDH-bright populations with no significant effects on 2D bulk cell culture, and blocked the growth of MEC xenografts

**Fig. 2:**
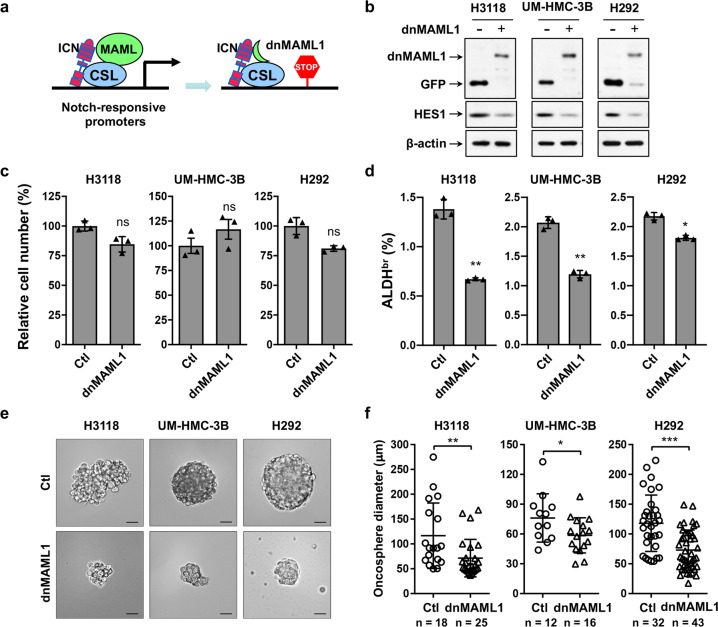
Expression of a dominant-negative MAML1 reduced the ALDH-bright and oncosphere-forming MEC cell population. **a** A schematic depicts the mechanism of dominant-negative MAML1 (dnMAML1) -mediated pan-Notch inhibition. **b** Human MEC cells (H3118, UM-HMC-3B and H292) were stably transduced with either pMSCV-dnMAML1 [MAML1(13-74 aa)-GFP] or pMSCV-GFP retroviruses and Western blot analysis was performed. An anti-GFP antibody was used to detect dnMAML1 expression in immunoblotting. **c** Cell proliferation assays were performed for dnMAML1-transduced vs the control GFP-transduced MEC cells (*n* = 3; ns, *p* > 0.05). **d** The percentages of ALDH^br^ cells in control GFP- and dnMAML1-expressing human MEC cells were assayed using an AldeRed ALDH Detection kit (*p < 0.05, **p < 0.01). **e** Representative images of oncospheres from control GFP and dnMAML1-expressing cells were shown (Bar = 50 µm). **f** Individual oncosphere size of each sample from 1000 initial cells was shown (*p < 0.05, **p < 0.01, ***p < 0.001)

To investigate the impact of Notch signaling inhibition on MEC growth, we first used a dominant-negative MAML1 (dnMAML1), which was previously characterized as a pan-Notch inhibitor.^38^ The dnMAML1 contains the Notch binding domain of the MAML1 transcriptional co-activator (13–74 aa) fusing to GFP and lacks the transcription activation domain. The dnMAML1 competes with the endogenous MAML coactivators and forms inert transcriptional complexes, thus blocking transcription from all 4 Notch receptor-mediated signaling (Fig. 2a). This dnMAML1 was shown to be a useful tool for probing the role of Notch signaling in various developmental and disease contexts in vitro and in vivo.^39–41^ We transduced human MEC cells (H3118, UM-HMC-3B and H292) with retroviruses expressing dnMAML1 or control GFP, FACS-sorted for GFP-positive cells, and assayed them for dnCRTC and HES1 expression, cell proliferation, aldehyde dehydrogenase (ALDH)-positive cells, and oncosphere formation. Western blot analysis validated dnMAML1 or GFP expression (Fig. 2b) using anti-GFP antibodies and decreased level of the Notch target HES1. There was no significant difference in the overall cell numbers between dnMAML1-expressing and GFP-expressing control cells in the 2D cell culture (Fig. 2c). Since MEC cells with a high level of aldehyde dehydrogenase (ALDH-bright, ALDH^br^) were previously shown to be enriched with stem-like (tumor-initiating) cells,^22^ we determined whether dnMAML1 expression affected this population in human MEC cells. We performed AldeRed ALDH Detection assays with a red-shifted fluorescent substrate for ALDH that allows the concurrent use of green fluorescent reporters. We observed a reduction in ALDH-bright population in dnMAML1-expressing vs the control GFP-expressing MEC (2d). Moreover, we showed that the dnMAML1-expressing cells gave rise to spheres with significantly reduced sizes as compared with control cells when grown as suspensions in serum-free stem cell culture medium (Fig. 2e, f). Therefore, Notch signaling inhibition via dnMAML1 affected only those MEC cells that were capable of generating spheres and were ALDH-bright cells; these cells account for a very small population of MEC cells in the 2D culture, which could explain no significant change in the 2D bulk cell culture. These results indicated that Notch signaling is required to maintain sphere-forming cells that likely reflect MEC stem-like cells.

**Fig. 3:**
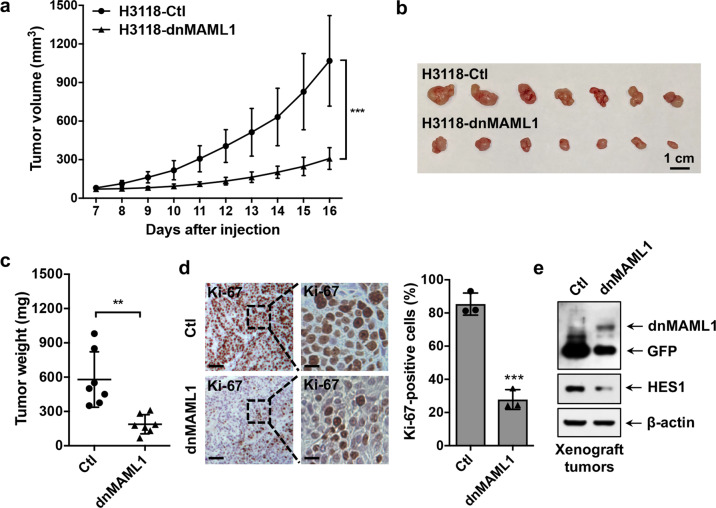
Expression of a dominant-negative MAML1 reduced the growth of MEC xenografts. GFP-expressing and dnMAML1-expressing H3118 cells (1 × 10^6^ cells/mouse) were subcutaneously injected to the right flanks of 8–12-week-old immune-deficient NOD.SCID mice (*n* = 7 each group). Mice were euthanized after a 16-day xenograft study. **a**–**c** The tumor volumes were measured daily after tumor cell injection (**a**), and tumor size (**b**) and tumor weight (**c**) were presented at the endpoint. **d** Representative images of Ki-67 staining of H3118-dnMAML1 and control H3118-GFP xenografts (left bar = 100 μm, right bar = 25 μm) were shown. ImageJ was used to analyze Ki-67-positive cells in IHC staining results. Sections of individual tumors (*n* = 3) in each group were analyzed. The data were presented as the percentage (%) of Ki-67 positive nuclei to the total nuclei. **e** The expression levels of GFP, dnMAML1, and HES1 in xenograft tumors were analyzed by western blotting (**p < 0.01; ***p < 0.001)

To determine the consequences of dnMAML1-mediated Notch signaling inhibition on MEC growth in vivo, we subcutaneously implanted the same number of dnMAML1-expressing H3118 MEC cells or GFP-expressing control cells to NOD.SCID mice and monitored tumor growth. The dnMAML1 expression significantly attenuated the growth of H3118 MEC xenografts, as demonstrated by reduced tumor volume over time (Fig. 3a), reduced tumor size (Fig. 3b), and weight (Fig. 3c) at the endpoint. The immunohistochemical analysis showed a reduced number of Ki-67+ proliferative cells in dnMAML1-expressing as compared to the control tumors (Fig. 3d). Western blot analysis confirmed dnMAML1 expression and a reduced level of Notch target HES1 in dnMAML1-expressing xenograft tumors in comparison with GFP-expressing control tumors (Fig. 3e). These in vivo data as well as the in vitro results indicated that Notch signaling inhibition suppresses the growth of MEC tumors likely through inhibiting the MEC stem-like cells, suggesting that Notch signaling is required for maintaining MEC stem-like cells and tumor growth.

### Treatment of a γ-secretase inhibitor reduced the ALDH-bright population with no significant effects on the bulk MEC cells in 2D cell culture, and blocked the growth of the MEC xenograft tumors

**Fig. 4:**
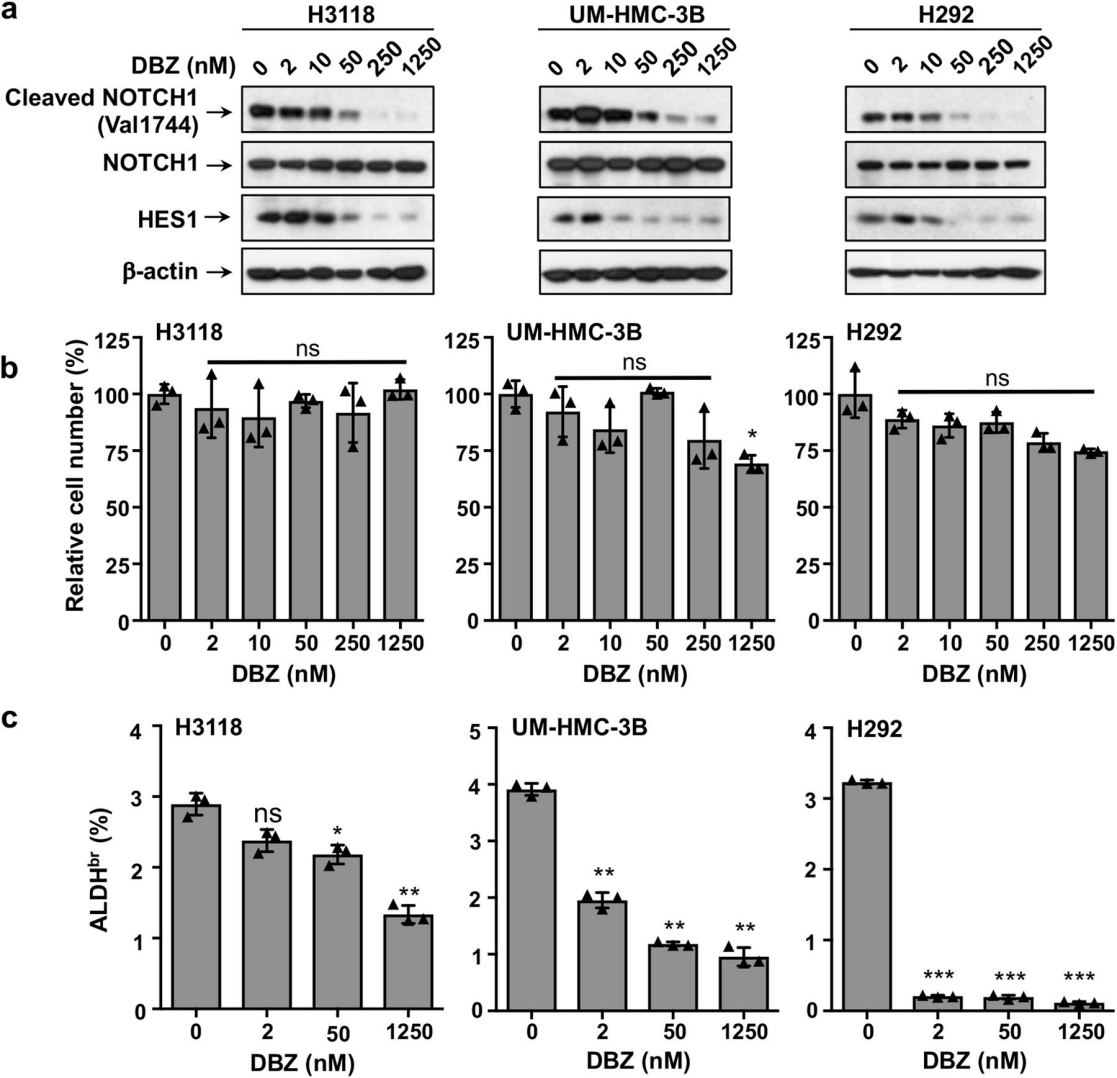
Treatment with the γ-secretase inhibitor DBZ reduced the ALDH-bright population in MEC cells. **a** Human MEC cells (H3118, UM-HMC-3B, and H292) were treated with various concentrations of DBZ for 72 h. The DBZ-treated cells were harvested for western blotting to analyze the levels of activated form of NOTCH1 (cleaved NOTCH1), the total NOTCH1, and HES1 proteins. **b** The number of the DBZ-treated cells were scored. **c** The DBZ-treated cells were assayed for the percentage of ALDH^br^ cells using an ALDEFLUOR™ Kit (ns, *p* > 0.05; *p < 0.05; **p < 0.01; ***p < 0.001)

We next tested the effect of a pharmacological Notch inhibitor, a γ-secretase inhibitor (GSI; DBZ) on MEC cell growth. GSI interferes with Notch receptor processing, preventing the release of the intracellular domain of Notch (ICN) from the cell membrane and blocking the transcriptional activation of Notch target genes. We first treated human MEC cells (H3118, UM-HMC-3B, and H292) with various concentrations of DBZ ranging from 0 to 1.25 μM for 72 h and observed a dose-dependent reduction of cleaved NOTCH1 (Val1744) and HES1 by western blot analysis, confirming that DBZ inhibited Notch signaling (Fig. 4a). Consistent with the effects of dnMAML1-mediated Notch inhibition, GSI treatment had no significant effects on the overall proliferation of MEC cells in the 2D culture, except for the highest dose of DBZ (1.25 µM) that caused a moderate decrease in the number of UM-HMC-3B cells (1.25 µM, *p < 0.05) (Fig. 4b). We also performed ALDEFLUOR assays and observed that the ALDH^br^ population accounted for about 3–4% of cultured MEC cells (H3118, UM-HMC-3B, and H292). There was a dose-dependent reduction in the ALDH^br^ MEC population after DBZ treatment for 72 h with the highest sensitivity to a low dose of DBZ in H292 cells (2 nM, ***p < 0.001) (Fig. 4c). Therefore, these data further support that the Notch signaling inhibition specifically targets a small stem-like cell population in the bulk MEC cell culture.

**Fig. 5:**
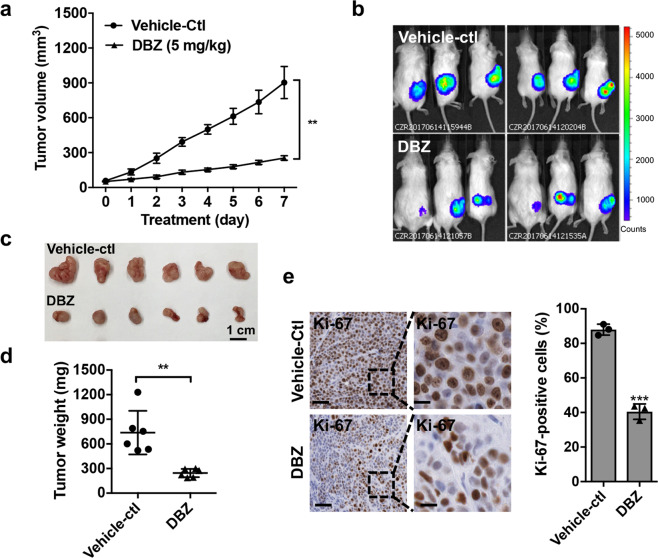
The γ-secretase inhibitor DBZ suppressed the growth of MEC xenografts. **a** NOD.SCID mice were subcutaneously injected with luciferase-expressing H3118 MEC cells. When the xenografts grew to ~50 mm^3^, mice were randomly separated and treated with either vehicle only (*n* = 6) or 5 mg/kg DBZ (*n* = 6) via intraperitoneal injection daily for 7 days. The tumor volumes were measured daily after treatment (**p < 0.01). **b**–**d** Bioluminescent imaging of the xenograft tumors (**b**), tumor size (**c**), and tumor weight (**d**) were presented on the final day of treatment. **e** Representative images showed Ki-67 immunohistochemical staining of H3118-luc xenografts treated with either vehicle control or DBZ (left bar = 100 μm, right bar = 25 μm). ImageJ was used to analyze Ki-67-positive cells on the sections of individual tumors (*n* = 3) in each group (**p < 0.01; ***p < 0.001)

We further assessed the effect of γ-secretase inhibitor DBZ on the growth of human MEC xenografts. NOD.SCID mice were subcutaneously implanted with firefly luciferase-expressing H3118 MEC cells (H3118-luc). When the average tumors reached 50 mm^3^, mice were randomly separated into two groups (*n* = 6 each) and treated with vehicle only or DBZ (5 mg/kg) daily via intraperitoneal injection. We observed significant tumor suppression in DBZ-treated vs. vehicle-treated mice, as shown by the size, volume, and weight of xenograft tumors (Fig. 5a–d). The Ki-67 IHC staining showed fewer proliferating cells in DBZ-treated xenograft tumors (Fig. 5e). These data indicate that the GSI (DBZ) significantly inhibited MEC growth in vivo. Combined with the in vitro data that GSI affected a small, potential MEC stem-like cell population while having a moderate effect on the growth of bulk human MEC cells, these data support that Notch inhibition via GSI treatment suppressed the growth of human MEC xenograft tumors by blocking the tumorigenic MEC stem-like cell population.

### The combination of the Notch inhibitor DBZ and the EGFR inhibitor Erlotinib inhibited the growth of MEC in vivo

Our above data showed that Notch signaling is important in maintaining MEC tumorigenic stem-like cells and that Notch receptor activation is independent of the CRTC1-MAML2 fusion. Therefore, we reasoned that simultaneous targeting of two independent signaling pathways in MEC, Notch signaling critical for MEC stem-like cells, and EGFR signaling driven by the major CRTC1-MAML2 oncogenic fusion, could achieve greater efficacy in blocking MEC.

**Fig. 6:**
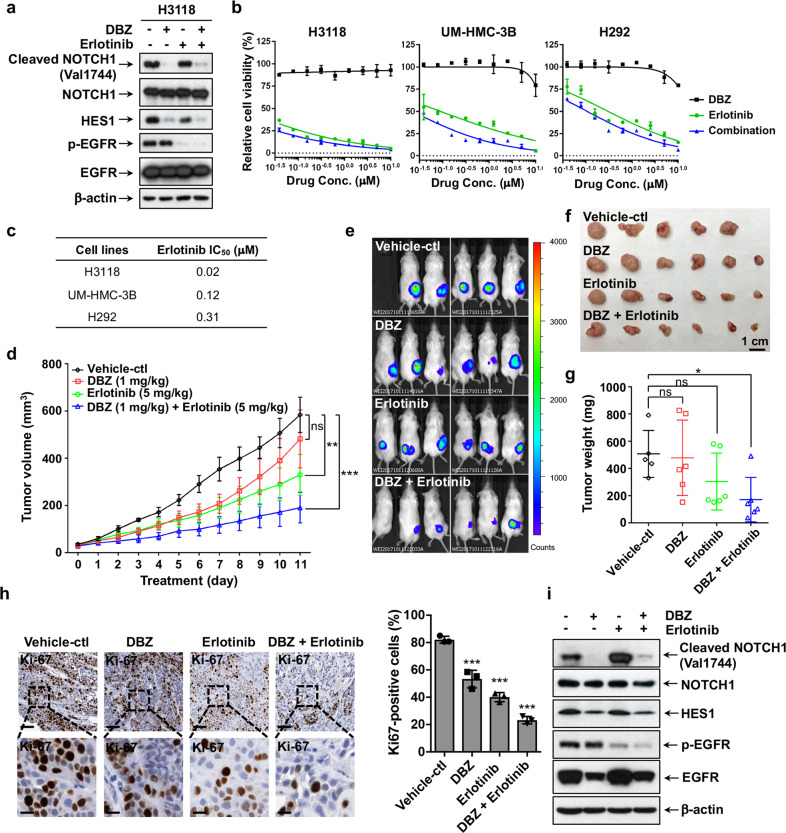
Co-inhibition of Notch and EGFR signaling synergistically inhibited the growth of MEC xenografts. **a** H3118 cells were treated with either DBZ (1 μM), Erlotinib (1 μM), or in combination for 24 h, and the treated cells were analyzed for Notch and EGFR signaling status by Western blot analysis using the antibodies as indicated. **b** Cell viability assays were performed on human MEC cells treated with various concentrations of DBZ, Erlotinib or in combination for 72 h. **c** The IC_50_ values of Erlotinib were shown. The IC_50_ of DBZ was not applicable as no inhibitory effect was observed. **d**–**g** NOD.SCID mice were subcutaneously inoculated with luciferase-expressing H3118 MEC cells. When the xenografts grew to ~50 mm^3^, mice were treated with vehicle control (*n* = 5), 1 mg/kg DBZ (*n* = 6), 5 mg/kg Erlotinib (*n* = 6), or 1 mg/kg DBZ plus 5 mg/kg Erlotinib (*n* = 6) via IP injection (DBZ) or oral gavage (Erlotinib) daily for 11 days. The tumor volumes were measured daily after treatment (**d**). Bioluminescent imaging of the xenograft tumors (**e**), tumor size (**f**), and tumor weight (**g**) were presented on the final day of treatment. **h** Representatives of Ki-67 staining of H3118-luc xenografts from each treatment group were shown (upper bar = 100 μm, lower bar = 25 μm). ImageJ was used to calculate Ki-67-positive cells in IHC staining results and sections of individual tumors (*n* = 3) in each group were analyzed. **i** Western blotting was performed to determine the levels of cleaved and total NOTCH1, HES1 as well as the phosphorylated and total EGFR post-drug treatments. The mice were given a boost administration of DBZ (5 mg/kg) and Erlotinib (25 mg/kg) either alone or in combination 2 h prior to euthanasia (*p < 0.05; **p < 0.01; ***p < 0.001)

We first tested the effects of the γ-secretase inhibitor DBZ and EGFR tyrosine kinase inhibitor Erlotinib, individually and in combination, on Notch signaling and EGFR signaling in human H3118 MEC cells. Western blot analysis showed that DBZ and Erlotinib blocked active Notch signaling and EGFR signaling, respectively, as evidence by reduced cleaved Notch 1 and p-EGFR, demonstrating their on-target activity (Fig. 6a). Interestingly, HES1 expression was also moderately reduced by Erlotinib, suggesting potential crosstalk of EGFR and Notch signaling. Next, we examined the viability of human MEC cells (H3118, UM-HMC-3B and H292) after the treatment of DBZ and Erlotinib, either alone or in combination (ratio 1:1) at various doses (Fig. 6b). The IC_50_ values (Fig. 6c) indicated that MEC cells, especially H3118 cells, were highly sensitive to EGFR signaling inhibition by Erlotinib. Notch inhibition by γ-secretase inhibitor (DBZ) did not affect the viability of the bulk MEC cells, and the combination of DBZ and Erlotinib only slightly enhanced the effect comparing to Erlotinib mono-treatment (Fig. 6b), which was consistent with our data that DBZ only affected a minor population in the 2D cell culture.

We next investigated the anti-MEC activity of DBZ and Erlotinib individually and in combination in vivo. Mice were subcutaneously injected with human H3118 MEC cells (H3118-luc) and were randomly divided into four groups when the xenograft tumors reached about 50 mm^3^. These mice were treated with vehicle only, DBZ (1 mg/kg, IP), Erlotinib (5 mg/kg, oral gavage), and DBZ (1 mg/kg) plus Erlotinib (5 mg/kg) daily for 13 days. Note that in this experiment we only administered about one-fifth dose of DBZ that was used in our previous experiment (Fig. 5) and about one-tenth dose of Erlotinib that was used in published tumor studies. The drug doses were selected because we aimed to determine whether anti-tumor efficacy can be achieved with low doses of drugs, consequently reducing drug toxicity, especially for Notch inhibitors with known on-target gastrointestinal toxicity.^42^ At these dose levels, DBZ showed no significant effect, while Erlotinib exhibited significant inhibition of MEC growth. Importantly, DBZ (1 mg/kg) plus Erlotinib (5 mg/kg) caused significantly better anti-tumor responses than either mono-therapies, as shown by the tumor volume over time, bioluminescence signal intensity from the tumors, and tumor weight (Fig. 6d, e, f, g). Mice were given a boost administration of DBZ (5 mg/kg) and Erlotinib (25 mg/kg) either alone or in combination 2 h prior to the tumor collection. Compared to vehicle control and each inhibitor alone, DBZ (1 mg/kg) plus Erlotinib (5 mg/kg) caused the greatest reduction in the proliferating tumor cells in xenograft tumors as shown by Ki-67 staining (Fig. 6h). Western blotting confirmed that DBZ reduced the levels of the active NOTCH1 (cleaved NOTCH1) and its target HES1 protein and that Erlotinib lowered the EGFR phosphorylation level in H3118 xenografts (Fig. 6i), suggesting that tumor suppression was caused by on-target effects of each drug. These data indicate that the combination of the Notch inhibitor DBZ and the EGFR inhibitor Erlotinib enhanced MEC suppression as compared to individual treatment.

## Discussion

Advanced, metastatic, and recurrent MECs have poor outcomes and no approved systemic therapy is currently available. Our previous research has shown that the CRTC1-MAML2 fusion, a potential major oncogenic driver in MEC, induced autocrine AREG-EGFR signaling that supports MEC cell growth and survival, thus revealing anti-EGFR therapies as a potential anti-MEC strategy.^11^ Due to the common resistance associated with anti-EGFR therapies, identifying and blocking other critical signaling for tumor growth will have the potential in enhancing tumor responses and reducing resistance. Despite mounting evidence supporting that MEC is likely maintained by a tumorigenic stem-like population, the molecular regulation of MEC stem-like cells remained poorly characterized. In this study, we revealed an essential role of Notch signaling in maintaining MEC stem-like cell compartment and provided evidence for co-targeting the stem cell pathway Notch signaling and CRTC1-MAML2-induced EGFR signaling as a potential effective anti-MEC treatment approach.

Published studies have shown that multiple cancer types contain cancer stem-like cells, a subpopulation of highly tumorigenic cells that are responsible for tumor initiation, maintenance, and metastasis.^43,44^ Several lines of evidence strongly support that MEC is maintained by stem/progenitor cells. First, MEC cells displayed histological features of cytokeratin patterns similar to normal salivary gland stem cells which reside in the ductal areas.^17–21^ Second, the CRTC1-MAML2 fusion was detected in all three major cellular components within MEC tumors, suggesting that salivary gland stem/progenitor cells after the initiation of the CRTC1-MAML2 oncogenic fusion event differentiate to various cellular components in human MEC. Third, a small cell population with high levels of ALDH activity and CD44 expression was present in human MEC cell lines, such as UM-HMC-3A and -3B, which displayed sphere-forming ability in vitro and tumorigenic potential in vivo.^22^ Fourth, human MEC cells appeared morphologically homogeneous in 2D culture, but were able to generate xenograft tumors with the typical MEC cell components, suggesting that MEC cell cultures contain a stem-like cell population capable of propagating the MEC tumor in vivo.^45^ Last, patients with MEC frequently exhibit loco-regional recurrence, regional and distant metastases, and lack of response to chemotherapy, which indicates that a stem-like cell population may contribute to MEC recurrence, metastasis, chemo- and radio-resistance.^23,24,46,47^ Therefore, stem-like cells likely drive MEC tumorigenesis. A better understanding of MEC stem cell regulation is critical for developing effective approaches for ablating MEC stem cells to prevent tumor recurrence and resistance to therapy.

Notch signaling plays a critical role in regulating cancer stem cells in multiple human cancers,^42,48^ but whether it has a role in regulating MEC had not been investigated. In this study, we utilized two approaches to inhibit endogenous Notch signaling in MEC cells, targeting the core Notch transcriptional complex with the dominant-negative MAML1 (dnMAML1) and interfering Notch receptor processing with γ-secretase inhibitors. While the inhibition of Notch signaling had no apparent effect on the proliferation of bulk MEC cells, it significantly reduced the oncosphere-forming and ALDH-bright populations in vitro and attenuated the growth of MEC xenografts in vivo. These data strongly demonstrate that Notch signaling critically contributes to the maintenance of a small subset of MEC stem-like cells that are capable of seeding tumor growth and suggest that targeting Notch signaling could effectively ablate cancer stem-like cells that could contribute to MEC recurrence. Activation of Notch signaling triggers expression of various target genes and we showed that inhibition of Notch signaling via dnMAML1 and the γ-secretase inhibitor (DBZ) reduced expression of a prototypic Notch target HES1. Moreover, HES1 expression can be regulated by the CRTC1-MAML2 fusion through a CREB-dependent mechanism, independent of Notch receptor activation. Therefore, HES1 expression is regulated by Notch signaling and the CRTC1-MAML2 fusion. It remains unknown whether HES1 or other Notch target genes mediate stemness in MEC. In the future, critical effectors for Notch signaling in maintaining the MEC stem-like cell phenotypes should be investigated.

Notch inhibitors have been under active clinical development.^49–51^ The γ-secretase inhibitors prevent the release of the active intracellular fragment of Notch and represent the major class of Notch inhibitors.^42^ Clinical testing of Notch inhibitors shows on-target intestinal toxicity (such as diarrhea); however, new approaches such as the adoption of intermittent dosing or the combination of Notch inhibitor and other drugs that reduce the dose of Notch inhibitor could potentially alleviate the toxicity while maintaining anti-tumor efficacy. Our results showed enhanced anti-MEC responses by targeting two independent signaling pathways in MEC, Notch signaling critical for MEC stem-like cells, and EGFR signaling driven by the major CRTC1-MAML2 oncogenic fusion. Moreover, effective anti-tumor responses were observed when the Notch inhibitor GSI (DBZ) and the EGFR inhibitor (Erlotinib) were used under low doses of individual inhibitors as compared to the doses normally used for the testing. Although the optimal doses and safety of the combination of GSI (DBZ) and the EGFR inhibitor (Erlotinib) remain to be further tested, our data strongly support that this combination is a promising therapeutic approach for MEC. EGFR and Notch signaling have fundamental roles during normal development and they frequently interact in cooperative or antagonistic manners.^52^ Complicated crosstalk between these two pathways in human cancers has been documented. For instance, EGFR blockade caused an enrichment of lung cancer stem-like cells through Notch-dependent signaling^53^ and co-targeting of these two pathways have shown anti-tumor efficacy with strong anti-stem cell effect.^54^ EGFR tyrosine kinase inhibitor-resistant lung cancer was also found to be responsive to the combined treatment of Notch inhibitors with EGFR inhibitors, gefitinib, or osimertinib.^55^ These studies further strengthen the concept of dual targeting of EGFR and Notch signaling in blocking tumor growth. It remains unclear how EGFR inhibition might affect Notch signaling in MEC, as we observed a reduced level of HES1 expression in MEC with the treatment of EGFRi. Moreover, the consequences of co-inhibition of Notch and EGFR in human MEC should be further elucidated at the molecular levels.

In summary, our study demonstrated a critical role of Notch signaling in maintaining MEC stem-like cells and tumor growth and revealed that the approach of co-targeting Notch and EGFR signaling is a potential effective anti-MEC treatment.

## Materials and methods

### Chemicals

The following chemicals were purchased from the commercial sources: the γ-secretase inhibitor (GSI) DBZ (CAS # 209984-56-5) from Cayman Chemical; the EGFR inhibitor Erlotinib hydrochloride (CAS # 183319-69-9) from LC laboratories; DMSO, methylcellulose and Tween-80 from Fisher Scientific; and Captisol from Ligand Pharmaceuticals.

### Plasmids

The retroviral constructs pMSCV-GFP and pMSCV-dnMAML1 [MAML1(13–74)-GFP] were previously described.^38^ The pMSCV-dnMAML1 construct expresses MAML1 (aa 13–74) fused to GFP under the control of the MSCV promoter.^38^ The lentiviral-based pLKO.1 shRNA constructs, shM2-1 (5′-CCCTGTCTAAACTCCAGGATA-3′) and shM2-3 (5′-CCCAAAGCAATTGTTAGCAAA-3′) were ordered from GE Dharmacon. The pLKO.1-scrambled shRNA control vector (#136035) and lentiCas9-Blast (#52962) were obtained from Addgene. The sgRNA sequences, sgMAML2-A (5′-TGTGAAGGACGATATGAACG-3′) and sgMAML2-B (5′-GATAGCACTGTGCACTCTCG-3′) targeting the exon 1 of the *MAML2* gene were designed using the CRISPR design tool (https://zlab.bio/guide-design-resources) and cloned into the lentiGuide-Puro vector (Addgene #52963).

### Cell culture, viral production, and transduction

H3118, UM-HMC-3A, UM-HMC-3B, and H292 are human MEC-derived cell lines. H3118 and H292 were of parotid and pulmonary origin, respectively. UM-HMC-3A, UM-HMC-3B, and UM-HPA-1 were generous gifts from Dr. Jacques Nör’s lab.^45^ UM-HMC-3A and UM-HMC-3B were derived from a local recurrence and metastatic lymph node from the same MEC patient, respectively. UM-HPA-1 was derived from a benign human pleomorphic adenoma. These cells as well as HEK293T and HEK293FT cells were cultured in Dulbecco’s modified Eagle’s medium (Corning Cellgro) supplemented with 10% fetal bovine serum (Gibco) and 1% penicillin-streptomycin (Corning Cellgro) at 37 °C with 5% CO_2_.

For retroviral production, HEK293T cells were transfected with pMSCV-based retroviral constructs, packaging plasmid pMD.MLV and envelope plasmid pMD2-VSV-G using the Effectene Transfection Reagent (Qiagen) per manufacturer’s instructions. For lentiviral production, HEK293FT cells transfected with lentiviral constructs, packaging plasmid psPAX2 and envelope plasmid pMD2.G using Effectene Transfection Reagent (Qiagen). Viral supernatants were harvested at 48 and 72 h post transfection. Cells were infected in two consecutive days by adding viral supernatant to fresh culture medium containing 8 μg/mL polybrene (Sigma) for 6 h.

### Quantitative RT-PCR

Total RNA was isolated using RNeasy Mini Kit (Qiagen #74106) and then reverse-transcribed into complementary DNA using a High Capacity cDNA Reverse Transcription Kit (Applied Biosystems #4368814). PCR was performed using the StepOne Real-Time PCR System with iTaq Universal SYBR Green Supermix (Bio-Rad #1725120). The relative gene expression was calculated using the comparative ΔΔCt method. Glyceraldehyde-3-phosphate dehydrogenase (GAPDH) was used as an internal control for normalizing gene expression among different samples. The following primer sequences are used for HES1 (5′-TCAACACGACACCGGATAAA-3′; 5′-TCAGCTGGCTCACACTTTCA-3′), MAML1 (forward, 5′-CACCAGCCACCGAGTAACTT-3′; reverse, 5′-CCCACAGTCCGCTTTGTAAT-3′); MAML2 (forward, 5′-TTTCCTTCACCCAACCAAAG-3′; reverse, 5′-GGGCCCATGTTATCATTTTG-3′); MAML3 (forward, 5′-CGTATATCCAGCAGCAGCAA-3′; reverse, 5′-TTTCTGGTCTTCGCTCAGGT-3′); and GAPDH (forward, 5′-CAATGACCCCTTCATTGACC-3′; reverse, 5′-GACAAGCTTCCCGTTCTCAG-3′).

### Western blotting

Western blotting analyses were performed as described previously.^56^ The following antibodies were used: anti-MAML2 (4618), anti-phospho-EGFR (Tyr 1068) (3777), anti-EGFR (2232), anti-cleaved-NOTCH1 (Val1744) (4147), anti-NOTCH1 (3608), and anti-HES1 (11988) from Cell Signaling Technology, and anti-β-actin (A5316) from Sigma-Aldrich.

### Cell proliferation assays

Human MEC cells transduced with pMSCV-dnMAML1 or pMSCV-GFP control retroviruses were seeded in 6-well plates at 0.5 × 10^6^ cells/well and cultured for 96 h. For the GSI DBZ treatment, cells were seeded in 6-well plate at 0.5 × 10^6^ cells/well overnight and treated with vehicle control (0.1% DMSO) or various concentrations of DBZ as indicated for 72 h. The cells were trypsinized and the number of viable cells were determined by using Trypan blue (Fisher scientific) exclusion assay. Triplicate assays were performed.

### Cell viability assays

Human MEC cells (2000 cells/well) were seeded in 96-well plates overnight and then treated with either vehicle only (0.1% DMSO), DBZ, Erlotinib or their fixed-ratio (1:1) combination at nine dosages (39 nM, 78 nM, 156 nM, 312 nM, 625 nM, 1.25 μM, 2.5 μM, 5 μM, 10 μM) for 72 h. Triplicate assays were set up. The cell viability was measured by using the Cell Titer-Glo luminescent cell viability assay kit (Promega) according to the manufacturer’s instructions. The IC_50_ values were calculated by GraphPad Prism 6.0 using non-linear regression analysis for inhibition dose-response.

### Oncosphere formation assays

Human MEC cells were seeded at 500 cells per well in 24-well ultralow-attachment plates (Corning) and cultured in serum-free DMEM/F12 medium supplemented with penicillin-streptomycin (1%), GlutaMAX (2 nM), human EGF (20 ng/mL), human FGF2 (20 ng/mL), N-2 supplement (1%) and insulin (10 µg/mL). All the growth factors were ordered from Sigma-Aldrich and other supplements were ordered from ThermoFisher. After 2-week culture, spheres were photographed under microscope (Leica). Two biological repeats (1000 initial cells in total) were set up and used for analysis. The oncosphere size was presented based on the diameter measured by ImageJ (version 1.51J8).

### ALDH detection assays

Human MEC cells were seeded in 6-well plates at 0.5 × 10^6^ cells/well overnight and then treated with vehicle (0.1% DMSO) or various concentrations of DBZ for 72 h. Cells were trypsinized (0.25% Trypsin without EDTA) and suspended in ALDEFLUOR Assay Buffer (STEMCELL Technologies) and subjected to ALDEFLUOR assays (#01700) following the manufacturer’s instructions. Flow cytometric analyses were performed using BD Accuri C6 cytometer (BD Biosciences). The 488 nm excitation laser was used to identify the ALDH^br^ cell population. AldeRed ALDH Detection Assay using a red-shifted fluorescent substrate for ALDH (Sigma, SCR150) was used for GFP-expressing or dnMAML1-expressing cells according to the manufacturer’s instructions.

### Mouse xenograft studies

Xenograft studies were performed using NOD.SCID mice (NOD.CB17-Prkdc^scid^/J, The Jackson Laboratory). For determining the effects of dnMAML1 expression on the growth of H3118 MEC xenografts, mice aged 8–12 weeks old were randomly divided into two groups, one injected with GFP-expressing H3118 cells (control) and the other with dnMAML1-expressing H3118 cells (dnMAML1). A total of 1 million cells suspended in 100 μL Matrigel (BD Biosciences)/PBS solution (*v*/*v* = 1:1) were subcutaneously injected to the right flank of each mouse. Tumors were measured daily using a Dial caliper and tumor volumes were calculated by the following formula: tumor volume = (length × width^2^) × 0.5.

For evaluating the in vivo anti-MEC efficacy of Notch inhibitor DBZ and/or EGFR inhibitor Erlotinib, we used firefly luciferase-expressing H3118 cells (H3118-luc) for growing MEC xenografts. A total of 1 × 10^6^ H3118-luc cells was subcutaneously injected to each mouse. When xenografts reached around 50 mm^3^, mice were randomly separated into 2 cohorts for drug treatment in one experiment (vehicle, DBZ) and 4 cohorts in another experiment (vehicle, DBZ, Erlotinib, combined drugs). Vehicle contained 2.3% DMSO in 0.5% methylcellulose, 0.1% Tween-80. DBZ (dissolved in 0.5% methylcellulose, 0.1% Tween-80) was given via intraperitoneal (IP) injection and Erlotinib (dissolved in 10% Captisol) was given via oral gavage. The body weight and tumors were measured daily. Bioluminescence pictures were taken before mice were euthanized. Mouse studies were carried out following a protocol that was approved by the Institutional Animal Care and Use Committee, the University of Florida.

### Immunohistochemistry (IHC)

The Ki-67 IHC staining was performed by the Molecular Pathology Core at University of Florida as previously described.^57^ Briefly, xenograft tumors were fixed in 4% paraformaldehyde followed by graded ethanol dehydration and paraffin embedding. The sections (4 µm) were de-paraffinized with xylene and re-hydrated using ethanol of decreasing concentration until only water was used. The activity of endogenous peroxidase was quenched with 3% hydrogen peroxide. Citra (Biogenex) was used to recover the antigenicity in samples and Background Sniper (Biocare Medical) was used for blocking. Slides were then incubated with a Ki-67 antibody (1:100, Dako, Agilent) for 60 min followed by a horse radish peroxidase (HRP) conjugated, goat anti-rabbit antibody (Biocare Medical) for 30 min. 3,3′-diaminobenzidine was applied to visualize the Ki-67 positive cells. The positively-stained nuclei were quantified using ImageJ.

### Statistical analyses

All statistical analyses were conducted using GraphPad Prism 7. Specifically, two-tailed student’s *t*-test was performed to analyze the difference of a variable in two groups. Multiple comparisons testing (Dunnett’s test) was performed to assess differences of a variable in three or more groups. Results with *p* value <0.05 were considered statistically significant.

## Data Availability

All the data that support the conclusions are presented in the paper. They also can be requested by contacting the corresponding author.
